# Activation of Intestinal Epithelial Stat3 Orchestrates Tissue Defense during Gastrointestinal Infection

**DOI:** 10.1371/journal.pone.0118401

**Published:** 2015-03-23

**Authors:** Nadine Wittkopf, Geethanjali Pickert, Ulrike Billmeier, Mousumi Mahapatro, Stefan Wirtz, Eva Martini, Moritz Leppkes, Markus Friedrich Neurath, Christoph Becker

**Affiliations:** 1 Department of Medicine 1, Friedrich-Alexander-University, 91052 Erlangen, Germany; 2 Institute of Translational Immunology, Johannes Gutenberg-University, 55131 Mainz, Germany; French National Centre for Scientific Research, FRANCE

## Abstract

Gastrointestinal infections with EHEC and EPEC are responsible for outbreaks of diarrheal diseases and represent a global health problem. Innate first-line-defense mechanisms such as production of mucus and antimicrobial peptides by intestinal epithelial cells are of utmost importance for host control of gastrointestinal infections. For the first time, we directly demonstrate a critical role for Stat3 activation in intestinal epithelial cells upon infection of mice with *Citrobacter rodentium* – a murine pathogen that mimics human infections with attaching and effacing *Escherichia coli*. *C*. *rodentium* induced transcription of IL-6 and IL-22 in gut samples of mice and was associated with activation of the transcription factor Stat3 in intestinal epithelial cells. *C*. *rodentium* infection induced expression of several antimicrobial peptides such as RegIIIγ and Pla2g2a in the intestine which was critically dependent on Stat3 activation. Consequently, mice with specific deletion of Stat3 in intestinal epithelial cells showed increased susceptibility to *C*. *rodentium* infection as indicated by high bacterial load, severe gut inflammation, pronounced intestinal epithelial cell death and dissemination of bacteria to distant organs. Together, our data implicate an essential role for Stat3 activation in intestinal epithelial cells during *C*. *rodentium* infection. Stat3 concerts the host response to bacterial infection by controlling bacterial growth and suppression of apoptosis to maintain intestinal epithelial barrier function.

## Introduction

Bacterial infections of the gastrointestinal tract are a frequent cause of diarrhea followed by dehydration and are estimated to cause 2.5 million deaths per year, especially in underdeveloped countries [1]. Among the diarrhea-inducing bacteria, pathogenic *Escherichia coli* such as enteropathogenic *E*. *coli* (EPEC) and enterohaemorrhagic *E*. *coli* (EHEC) frequently cause outbreaks of diarrheal diseases [2, 3]. The largest outbreak of EHEC infection ever seen in Europe occurred in Germany in spring 2011: more than 2,900 cases of acute gastroenteritis, more than 850 cases of haemolytic uraemic syndrome (HUS) and at least 50 related deaths in three months were listed [4].

EHEC and EPEC infections cause non-specific gastroenteritis and the benefit of antibiotic agents for treatment of EHEC and EPEC infections is a long-standing matter of debate [5]. Not only might current antibiotics shape the natural course of the disease, but affect the pathogen as well as the beneficial commensal gut flora. Accordingly, there is an urgent need for new therapeutic approaches to combat pathogens and prevent common side-effects such as antibiotic-associated diarrhea. Interestingly, the gut itself also possesses antibacterial defense mechanisms such as antimicrobial peptides (AMPs) produced by specialized intestinal epithelial cells, designated Paneth cells [6, 7]. Therefore, studies make efforts to investigate the role of AMPs during infections with intestinal pathogens. For example, mice overexpressing the antimicrobial peptide CRAMP (cathelicidin-related antimicrobial peptide) were demonstrated to be protected from oral infection with *Citrobacter rodentium* (*C*. *rodentium*) [8], a naturally occurring mouse pathogen that is commonly used to mimic human infections with EHEC and EPEC.

The body’s own antimicrobial defense mechanisms in the gut are of utmost importance for maintenance of a healthy intestine. Using a mouse model of inflammatory bowel disease, we recently suggested a role of Stat3 (signal transducer and activator of transcription 3) activation in intestinal epithelial cells (IECs) for tissue homeostasis. Interestingly, among the genes which were differentially regulated in Stat3 deficient IECs, several were previously reported to show antimicrobial functions. Consequently, Gene Ontology analysis indicated that Stat3 might be involved in antimicrobial defense [9]. Stat3 is a transcription factor whose increased activation in colonic epithelial cells has been observed in some patients with active inflammatory bowel disease (IBD) [10, 11]. Moreover, genome-wide association studies have linked polymorphisms in the allele encoding Stat3 with an increased risk for the development of IBD [12, 13]. Stat3 is activated by binding of certain ligands such as IL-6, IL-10 and IL-11 to their specific receptors; activated Stat3 then transfers to the nucleus and induces the transcription of target genes including *BCL2*, *MYC* and *BIRC5*. It has recently been demonstrated that IL-22 induces activation of Stat3 and that IL-22 is crucial for production of AMPs in the intestine [14]. Accordingly, IL-22 is important to combat most intestinal infections. While IL-22 production is increased after infection with *Listeria monocytogenes*, it is not required for clearance of the infection [15]. Yet, IL-22 deficient mice develop severe colitis after infection with *C*. *rodentium* and show high mortality [14]. Therefore, although IL-22 expression is increased during colitis, IL-22 is hypothesized to exert protective functions via activation of Stat3 in the intestinal epithelium. Although IL-22 has been demonstrated to protect the host from gastrointestinal infections, the functional role of Stat3 in the epithelium has not been demonstrated. Using intestinal epithelial cell specific Stat3 deficient mice (Stat3^ΔIEC^), we demonstrate a critical role for Stat3 activation in IECs during *C*. *rodentium* infection. Our data show that mice unable to activate Stat3 in IECs are severely impaired in their ability to defend against *C*. *rodentium*. Mechanistical data indicate that Stat3 orchestrates the intestinal epithelial response to bacterial infection by production of antimicrobial peptides and suppression of apoptosis.

## Materials and Methods

### Mice

We obtained C57BL/6 wildtype mice from the animal facility of the University of Mainz. Mice carrying both loxP-flanked Stat3 alleles (Stat3^fl/fl^) and the Cre-recombinase under control of the Villin-promoter were described before (Stat3^ΔIEC^) [9]. All mice were housed in individually ventilated cages. To exclude an effect of different mouse microbiota in separated cages, Stat3^ΔIEC^ mice and control (Stat3^fl/fl^) littermates were housed in mixed cages. The protocol was carried out in strict accordance with the recommendations in the Guide for the Care and Use of Laboratory Animals of the National Institutes of Health. The protocol was approved by Landesuntersuchungsamt Koblenz (Permit Number: 2.3 177–07/G 07–1–006). Mouse endoscopy and IVIS analysis were performed using isoflurane anesthesia, and all efforts were made to minimize suffering.

### *Citrobacter rodentium* infection and endoscopy

For bacteria-induced colitis, mice were infected with an erythromycin resistant and luminescent strain of *Citrobacter rodentium* (*C*. *rodentium* strain ICC169 was kindly provided by C. Riedel [29]). Bacteria were grown in sterile LB-Medium supplemented with 500 μg / ml erythromycin at 37°C and resuspended in sterile PBS to a final concentration of approximately 4 x 10^9^ bacteria per 200 μl. Mice were fastened for eight hours and 200 μl of bacteria suspension were given to each animal using a feeding needle. For quantification of the applied numbers of *C*. *rodentium*, the bacterial suspension was plated on CASO-blood agar plates (Heipha Dr. Müller GmbH, Eppelheim, Germany). For monitoring of luminescent *C*. *rodentium* growth and distribution in live mice, mice were anesthetized by gassing with isoflurane (1.5–2% mixed with air; Abbott).The abdomen of the mice was depilated and luminescence was measured using an IVIS Lumina II system (Caliper Life Science, Waltham, Massachusetts, USA). Quantification of luminescence was performed using the IVIS-associated software “Living Image” (Caliper Lifescience). For follow up development of colitis and endoscopic scoring, the Coloview high resolution mouse endoscopic system (Storz, Tuttlingen, Germany) was used as previously described [30]. Health of mice was monitored by weighing mice every other day. At the end of the experiments, mice were sacrificed by cervical dislocation. Tissue samples were collected and either instantly frozen in liquid nitrogen or fixed in Histofix (Roth, Karlsruhe, Germany).

### Organoid culture

Small intestinal crypts were isolated and cultured as previously described by Sato et al [31]. After 6 days of organoid growth, organoids were treated with IL-22 (100 ng / ml; Peprotech, Rocky Hill, New Jersey, USA) for 24 hours or left untreated. Organoids were harvested, washed twice with PBS and embedded in Histogel (Thermo Scientific, Waltham, Massachusetts, USA) according to manufacturer’s recommendations. After solidifying, solid Histogel-organoid samples were fixed in Histofix (Roth).

### Expression analysis

mRNA was isolated using the NucleoSpin RNA II kit (Macherey-Nagel, Düren, Germany) and reverse transcribed into complementary DNA using the iScript cDNA Synthesis Kit (Bio-Rad, Hercules, California, USA) as recommended by the manufacture. Quantitative PCR was performed using cDNA-specific Quantitect Primer assays (Qiagen, Hilden, Germany) and SsoFast EvaGreen Supermix (Bio-Rad) according to the manufacturer’s recommendations. Samples were either normalized to the general housekeeping gene *Hprt* or the intestinal epithelial housekeeping gene *Villin*.

### Histological examination

For histological examination, frozen or dehydrated and paraffin-embedded Histofix-fixed tissues were sectioned. Sections were stained with Mayer’s haematoxylin & eosin (H & E) as recommended by the manufacturer’s protocol. For immunohistochemical analysis, frozen tissue slices were fixed on glass slides using 4% PFA and paraffin-embedded tissue sections were dewaxed and rehydrated. anti-pStat3 (Cell Signaling, Danvers, Massachusetts, USA), anti-MPO (Abcam, Cambridge, United Kingdom), anti-CD4 (BD Pharmingen, San Jose, California, USA), anti-CD11c (BD Bioscience, San Jose, California, USA) and anti-RegIIIγ (antibodies online GmbH, Aachen, Germany) were used as primary antibodies. A biotinylated secondary anti-rabbit-antibody (Dianova, Hamburg, Germany) was used together with the TSA Cy3 system (Perkin Elmer, Waltham Massachusetts, USA) in accordance with the manufacturer’s protocol. Dying cells were detected using TUNEL assay (In situ Cell death Detection Kit Fluorescein, Roche, Basel, Switzerland) as recommended by the manufacturer. Bacteria present in tissues were detected by fluorescence *in situ* hybridization (FISH) of bacterial RNA as previously described [32]. Nuclei were counterstained with Hoechst (Invitrogen, Darmstadt, Germany). Images were obtained using the bright-field / fluorescence microscope Olympus IX70 (Olympus, Hamburg, Germany) or Leica DMI4000 B (Leica, Wetzlar, Germany).

### Statistical analysis

Statistical analysis was performed using the two-tailed student’s t-test. * p ≤ 0.05, ** p ≤ 0.01, *** p ≤ 0.001, n.s. = not significant. Percentages of cells in immunofluorescence pictures were calculated using ImageJ (National Institute of Health) [33].

## Results

**Fig 1:**
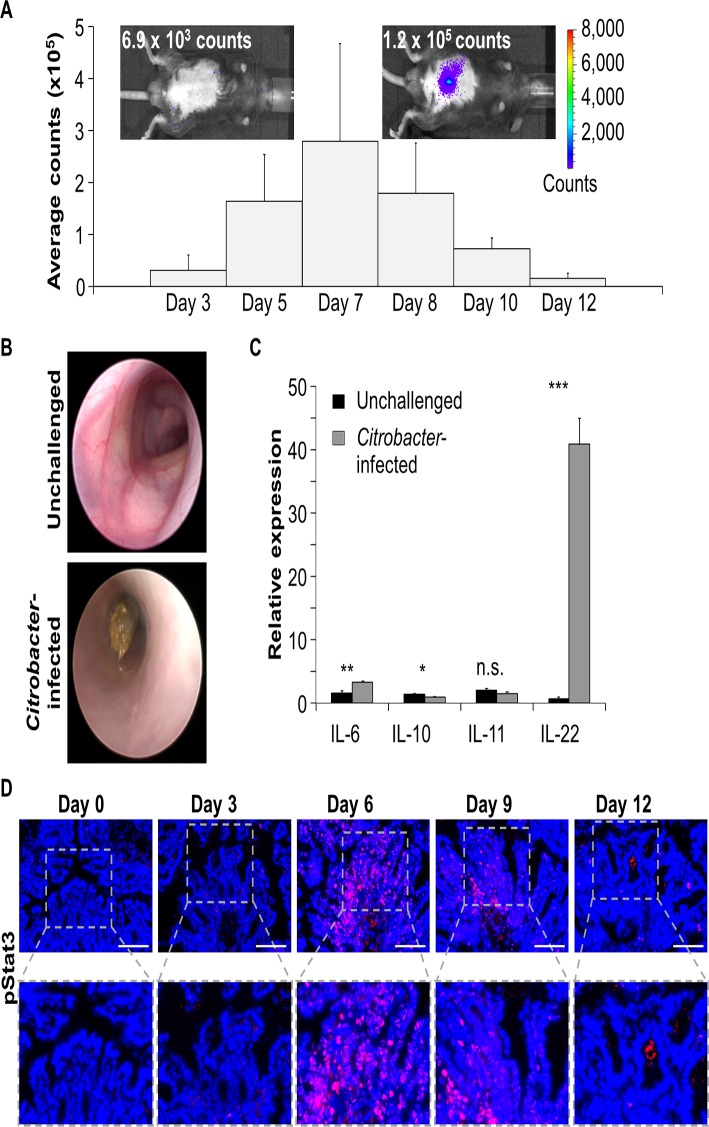
Infection of wildtype mice with *C*. *rodentium* activates Stat3 in the intestinal epithelium. A) Luminescent *C*. *rodentium* was visualized and quantified in live wildtype mice at different time points after infection using IVIS. Exemplary pictures on day three (left) and day seven (right) are shown. Data show mean values + SEM (n = 4 mice). B) Representative colonoscopy pictures demonstrate absence of colitis in unchallenged mice (top) and inflammation in the colon of *C*. *rodentium* infected mice (bottom, day six after infection). C) Transcription of Stat3-inducing cytokines in the colon of *C*. *rodentium* infected wildtype mice (n = 4) and unchallenged wildtype mice (n = 5). Gene transcription is normalized to *Hprt*. Data show mean values + SEM. D) Colon cross-sections of wildtype mice were stained for phosphorylated Stat3 (pStat3, red) at different time points of *C*. *rodentium* infection. Nuclei are shown in blue. Scale bars: 100 μm. The experiment was performed four times with similar results.

To examine the role of Stat3 for antimicrobial defense mechanisms during gastrointestinal infections *in vivo*, we used the *C*. *rodentium* induced infectious colitis mouse model. After oral infection of C57BL/6J wildtype mice with 4 x 10^9^ luminescent *C*. *rodentium*, the development of infection was monitored using an *in vivo* imaging system. In agreement with a transient infection, we observed a continuous increase in luminescence in the gut over seven days and a decrease to background levels afterwards (Fig. 1a). Endoscopic analysis of infected mice revealed signs of intestinal inflammation such as bowel-wall thickening, granularity of the mucosa, and fibrin formation (Fig. 1b). Upon screening for the expression of cytokines known to regulate intestinal defense mechanisms, IL-6 and IL-22 revealed strong induction after bacterial infection (Fig. 1c). Our finding is in line with recent data published by Zheng et al. [14], showing that IL-22 was strongly increased after *C*. *rodentium* infection. IL-22 has been reported to be produced by several cell types including dendritic cells [9], neutrophils [16], Th17 cells [17, 18] and subsets of NK cells [19, 20]. However, recent data suggest RORγt^+^ innate lymphoid cells (ILCs) to be the main producers of IL-22 in the gut [21]. Both IL-6 and IL-22 can induce the Stat3 signaling pathway in epithelial cells upon binding to their receptors. To analyze Stat3 activation in the intestinal epithelium in response to bacterial infections, gut samples from wildtype mice were harvested at different time-points and stained for phosphorylated Stat3. While colonic epithelial cells of uninfected mice showed no phosphorylated Stat3, the transcription factor was activated after infection with *C*. *rodentium* (Fig. 1d). Notably, the number of IECs showing activation of Stat3 increased with progression of infection. In striking similarity to *C*. *rodentium* derived luminescence signals, the strongest phosphorylation of Stat3 was observed around day 6. These results suggest activation of Stat3 as a biological response of the intestinal epithelium to bacterial infection.

**Fig 2:**
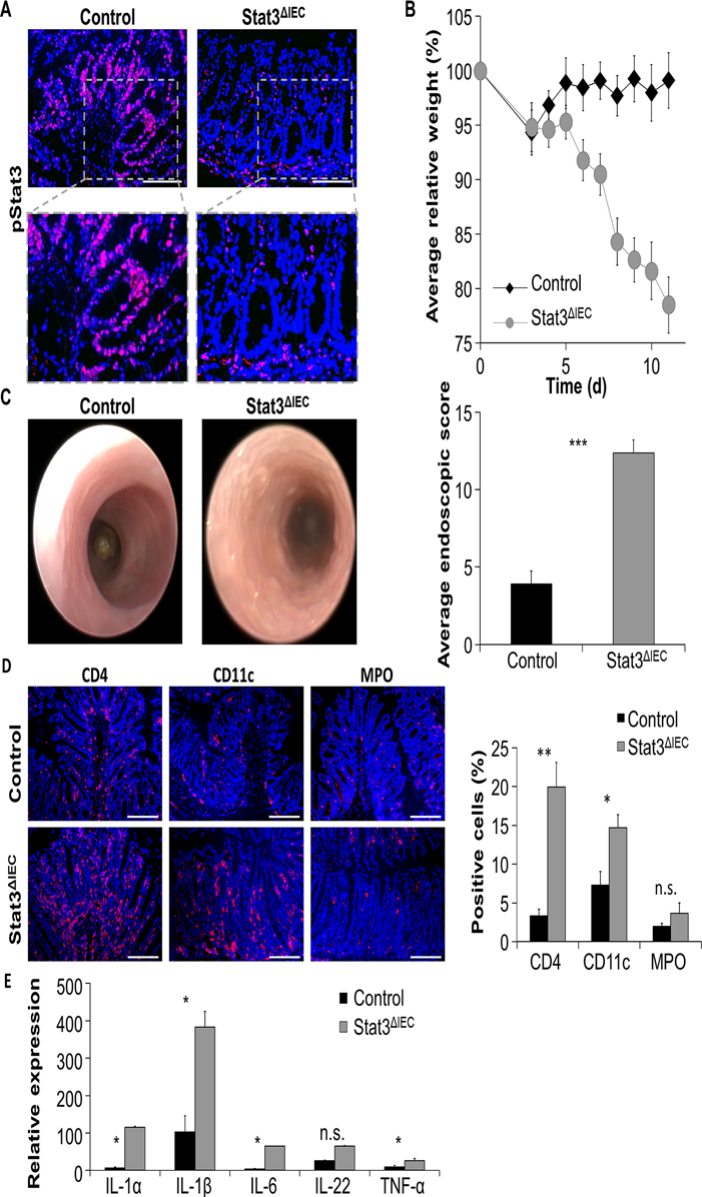
Stat3^ΔIEC^ mice are hypersusceptible to *C*. infection and develop severe colitis. A) Immunofluorescence staining detecting pStat3 (red) in colon cross-sections of wildtype and Stat3^ΔIEC^ mice infected with *C*. *rodentium* demonstrates absence of activated Stat3 in intestinal epithelial cells of Stat3^ΔIEC^ mice during infection with *C*. *rodentium*. Nuclei were counterstained with Hoechst (blue). Scale bars: 100 μm. B) Body weights of control and Stat3^ΔIEC^ mice were monitored during the course of infection and calculated relative to day 0. Data show mean values +/- SEM (n = 4 control mice, n = 6 Stat3^ΔIEC^ mice). C) Left: Endoscopic pictures of *C*. *rodentium* infected control and Stat3^ΔIEC^ mice show severe signs of colonic inflammation in infected Stat3^ΔIEC^ mice. Right: Severity of colitis was quantified by endoscopic scoring. Data show mean values + SEM (n = 3 mice in each group). D) Left: Representative pictures of colonic cross-sections derived from infected control and Stat3^ΔIEC^ mice stained for CD4 (red), CD11c (red) and MPO (red). Nuclei were stained with Hoechst (blue). Scale bars: 200 μm. Right: Software-based calculation of the proportion of CD4^+^ cells, CD11c^+^ cells, and MPO^+^ cells in immunofluorescence pictures stained for the designated cells. Data show mean values + SEM. E) Transcription of cytokines in the colon of *C*. *rodentium* infected control and Stat3^ΔIEC^ mice. Data were calculated relative to infected control and show mean values + SEM (n = 4 mice in each group). *Hprt* was used as internal standard. Four independent experiments were performed with similar results on each occasion.

For a direct functional analysis of Stat3 activation in intestinal epithelial cells during gastrointestinal infections, IEC-specific Stat3 deficient mice (Stat3^ΔIEC^) and control littermates (Stat3^fl/fl^) were challenged with *C*. *rodentium*. The efficiency of conditional Stat3 deletion was confirmed by histological staining of colon samples derived from infected control and Stat3^ΔIEC^ mice: while colonic tissue of infected control mice showed activated Stat3 in both lamina propria and colonic epithelial cells as demonstrated above, phosphorylated Stat3 was detectable in lamina propria cells but not IECs of infected Stat3^ΔIEC^ mice (Fig. 2a). IEC-specific Stat3 conditional knockout mice were highly susceptible to *C*. *rodentium* infection. Stat3^ΔIEC^ mice significantly lost more body weight than controls and showed a general deterioration in health of these animals, as evidenced by a lack of activity and ruffled fur (Fig. 2b). While control mice developed mild intestinal inflammation, infected Stat3^ΔIEC^ mice showed severe signs of colonic inflammation with high granularity, strong formation of fibrin and diarrhea (Fig. 2c). The difference in extent of colitis was confirmed by statistical analysis of endoscopic colitis scores (Fig. 2c). In agreement with endoscopic analysis, immunofluorescence stainings of gut cross-sections revealed prominent infiltrations of CD4 positive and CD11c positive immune cells into the lamina propria of infected Stat3^ΔIEC^ mice (Fig. 2d). Moreover, Stat3^ΔIEC^ mice showed increased expression of inflammatory cytokines such as IL-1α, IL-1β, IL-6 and TNF-α (Fig. 2e). Together, our results show that a defect in activating Stat3 in intestinal epithelial cells leads to the development of a strikingly more severe intestinal inflammation after infection with *C*. *rodentium* as compared to wildtype mice.

**Fig 3:**
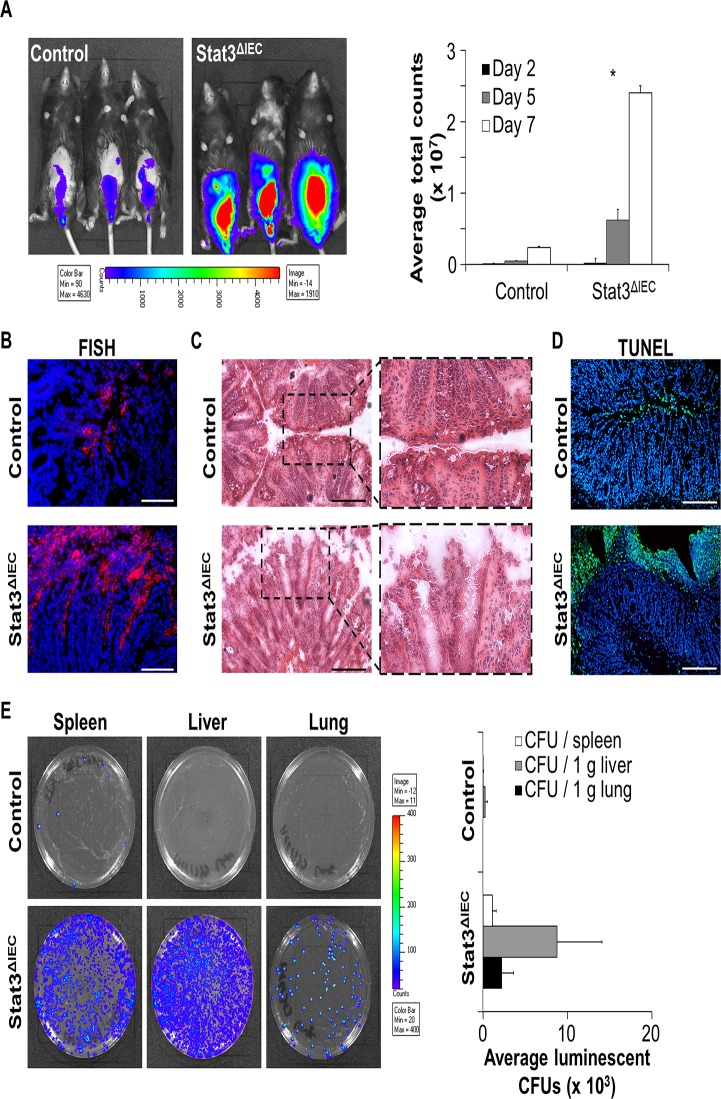
Breakdown of intestinal epithelial barrier in infected Stat3^ΔIEC^ mice facilitates systemic spread of *C*. *rodentium*. A) Left: Visualization of luminescent *C*. *rodentium* in live mice by IVIS on day six after infection. Right: Quantification of *C*. *rodentium* infestation by measuring *C*. *rodentium* luminescence at different time points after infection of control and Stat3^ΔIEC^ mice. Data show mean values + SEM (n = 5 mice in each group). B) Fluorescence *in situ* hybridization (FISH) of bacterial RNA (red) present in colonic cross-section. Nuclei are shown in blue. Scale bars: 50 μm. C) H&E staining of colonic cross-sections from infected control and Stat3^ΔIEC^ mice. Scale bars: 200 μm. D) Colon cross-sections derived from *C*. *rodentium* infected control and Stat3^ΔIEC^ mice were stained for cell death by TUNEL (green). Nuclei were counterstained with Hoechst (blue). Scale bars: 200 μm. E) *C*. *rodentium* infestation of different organs in infected control and Stat3^ΔIEC^ mice was assessed by plating organ lysates on agar plates (luminescent CFU indicate *C*. *rodentium* colonies) (left) and counting luminescent CFUs (right) the next day. Data show mean values + SEM (n = 5 control mice, n = 4 Stat3^ΔIEC^ mice). Representative pictures are shown.

In line with increased intestinal inflammation in Stat3^ΔIEC^ mice, we detected enlarged infection loads of *C*. *rodentium* by *in vivo* imaging in the gut of these mice (Fig. 3a). To investigate bacterial colonization of the gut in a more detailed fashion, we performed FISH (fluorescence in situ hybridization) of colonic cross-sections and observed an increased amount of bacteria directly adherent to the intestinal epithelial surface (Fig. 3b). Remarkably, in contrast to control mice, where bacteria remained on the surface of the epithelial brush border, bacteria in infected Stat3^ΔIEC^ deficient mice penetrated deeply into the crypts. These data suggest the inflammatory phenotype observed in infected Stat3^ΔIEC^ mice to be aggravated by the excessively increased amount of bacteria adherent to the bowel wall and indicate defects in intestinal epithelial barrier function.

In fact, we demonstrate a disrupted intestinal surface epithelium in the colon of infected Stat3^ΔIEC^ mice by H&E staining (Fig. 3c). Since *C*. *rodentium* can induce apoptosis in intestinal epithelial cell lines [22], we investigated whether *C*. *rodentium* infected Stat3^ΔIEC^ mice show increased epithelial cell death. TUNEL (TdT-mediated dUTP nick-end labeling) staining revealed excessive cell death of intestinal epithelial cells in *C*. *rodentium* infected Stat3^ΔIEC^ mice (Fig. 3d). Cell death predominantly occurred within the surface epithelium of *C*. *rodentium* infected Stat3^ΔIEC^ mice directly exposed to the lumen, suggesting that Stat3 is required to control *C*. *rodentium* induced epithelial cell death and to maintain barrier integrity.

A serious complication of human gastrointestinal infections is systemic spread. To investigate whether the proposed barrier defects lead to translocation of bacteria from the lumen into the bowel wall and to distant sites, we harvested spleen, liver, and lung tissue and plated homogenized samples on LB-agar plates. Although *C*. *rodentium* has been reported to be a non-invasive pathogen [23], invasion of *C*. *rodentium* into all investigated organs of Stat3^ΔIEC^ mice was observed (Fig. 3e). In line with this observation, Stat3^ΔIEC^ mice not only showed severe intestinal inflammation but signs of sepsis such as dramatic weight loss, ruffled fur and lack of physical activity.

Collectively, these data demonstrate the essential requirement of Stat3 activation in IECs for the control of *C*. *rodentium* growth and for maintaining intestinal epithelial barrier function to prevent systemic spreading of bacteria.

**Fig 4:**
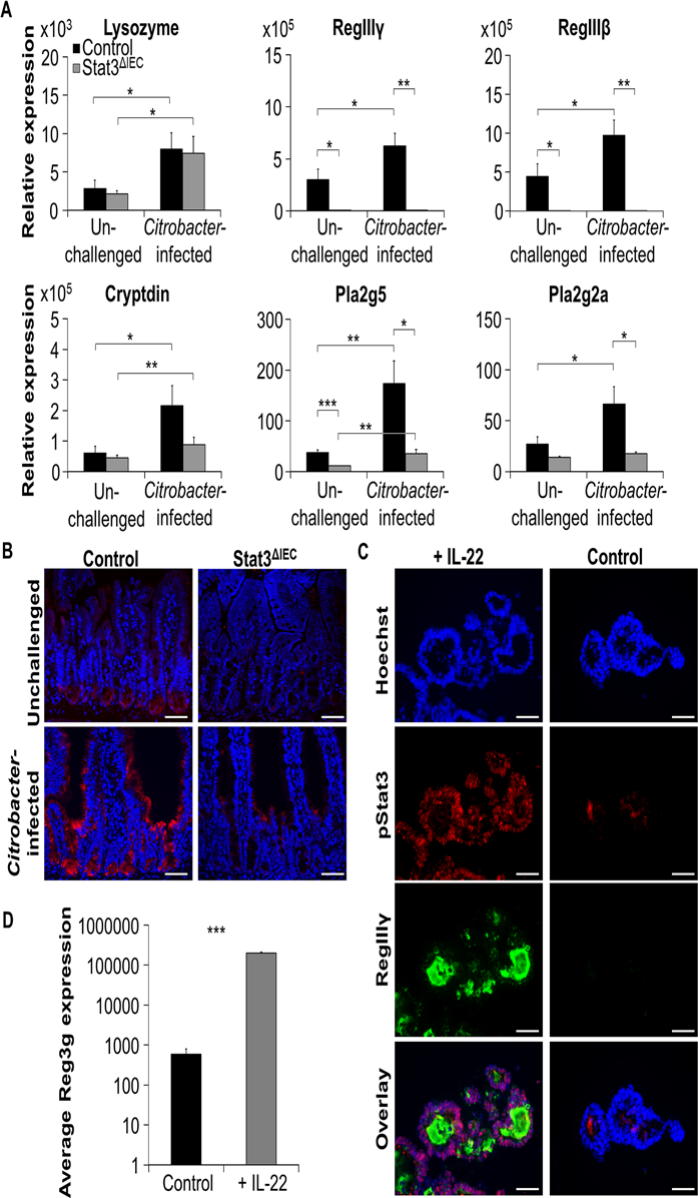
Impaired expression of AMPs in Stat3^ΔIEC^ mice. A) Quantitative transcription analysis was performed for small intestine of unchallenged control and Stat3^ΔIEC^ mice and *C*. *rodentium* infected (eight days after infection) control and Stat3^ΔIEC^ mice. Transcription of the antimicrobial peptides lysozyme, RegIIIγ, RegIIIβ, cryptdin, Pla2g5 and Pla2g2a was normalized to *Hprt* or *Villin*. Data show mean values + SEM (n = 5 unchallenged control mice, n = 5 unchallenged Stat3^ΔIEC^ mice, n = 5 *C*. *rodentium* infected control mice, n = 4 *C*. *rodentium* infected Stat3^ΔIEC^ mice). B) Representative pictures of small intestinal cross-sections derived from unchallenged and infected control and Stat3^ΔIEC^ mice were stained for RegIIIγ (red). Nuclei were stained with Hoechst (blue). Scale bars: 50 μm. C) Representative pictures of wildtype organoids treated with or without IL-22 for 24 hours and stained for pStat3 (red) and RegIIIγ (green) using immunohistochemistry. Nuclei were stained with Hoechst (blue). Scale bars: 50 μm. D) Transcription of RegIIIγ in organoids treated with IL-22 for 24 hours or left untreated (n = 6 from 3 different experiments). Gene transcription is normalized to *Hprt*. Data show mean values + SEM.

The increased population of *C*. *rodentium* in Stat3^ΔIEC^ mice suggested an impaired antimicrobial defense in the gut of Stat3^ΔIEC^ mice. We therefore examined the expression of antimicrobial peptides such as RegIIIγ, RegIIIβ, lysozyme, cryptdin, Pla2g5 and Pla2g2a in the gut of control and Stat3^ΔIEC^ mice, under unchallenged conditions and after infection with *C*. *rodentium*. Interestingly, unchallenged Stat3^ΔIEC^ mice lacked expression of RegIIIγ and RegIIIβ in the gut and showed significantly decreased transcription of Pla2g5 compared to unchallenged control mice (Fig. 4a). Infection with *C*. *rodentium* significantly induced the transcription of all the investigated antimicrobial peptide genes in the small intestine of control mice. However, induction of most AMPs was abrogated in infected Stat3^ΔIEC^ mice (Fig. 4a). Since RegIIIγ was recently described to promote host defense against *C*. *rodentium*, we next performed immunohistochemistry to investigate RegIIIγ expression in control and conditional Stat3 deficient mice. RegIIIγ was predominantly expressed at the crypt bottom of wildtype mice but was also detected in enterocytes and its expression strongly increased after infection with *C*. *rodentium* (Fig. 4b). In contrast, almost no expression of RegIIIγ in the gut of Stat3^ΔIEC^ mice was observed under unchallenged conditions and only small amounts were detected after infection (Fig. 4b). Thus, we hypothesize that Stat3 activation in IECs may drive the expression of AMPs and thereby controls growth of luminal bacteria.

We recently demonstrated Stat3 in intestinal epithelial cells to be activated via stimulation with IL-22 [9]. In agreement with the IL-22—pStat3—pathway driving RegIIIγ expression, phosphorylated Stat3 was detected in organoid epithelial cells simultaneously with high amounts of secreted RegIIIγ after IL-22 treatment (Fig. 4c). In fact, RegIIIγ expression in epithelial organoids was more than 300 fold increased after treatment with IL-22 (Fig. 4d). These data clearly show that IL-22 induces phosphorylation of Stat3 in intestinal epithelial cells followed by production of RegIIIγ.

The data obtained in the present study show that a defect in Stat3 activation leads to an impaired transcription of antimicrobial peptides thereby facilitating overgrowth of *C*. *rodentium*. The activation of Stat3 in the intestinal epithelium via IL-22 maintains the epithelial barrier function under stress conditions and prevents translocation of *C*. *rodentium* to systemic sites.

## Discussion

Intestinal infections lead to production of various cytokines and chemokines by lamina propria immune cells. The secreted cytokines not only influence other immune cells but provide constant information about the gut health status to the epithelial barrier and induce adequate epithelial responses [24]. Maintenance of intact intestinal epithelial barrier function is known to be crucial for combating intestinal infections. Recently, IL-22 has been found to play a major role in infections with the EHEC/EPEC like pathogen *C*. *rodentium* in mice [14]. In line with this report, we also demonstrated strong induction of IL-22 in the gut of mice infected with *C*. *rodentium*. However, our data go beyond the role of IL-22 and for the first time directly demonstrate a functional role of Stat3 signaling in intestinal epithelial cells for control of gastrointestinal infections *in vivo*. Our study collectively shows that epithelial Stat3 concerts the host response to bacterial infection by controlling bacterial growth and suppression of apoptosis to sustain intestinal epithelial barrier function. Interestingly, while our data together with the study published by Zheng et al. demonstrate the IL-22—Stat3 axis to be important in combating *C*. *rodentium* infections, IL-22 was shown to be detrimental in case of *Salmonella enterica serovar Typhimurium* infection [25]. It was concluded that IL-22 induced upon *S*. *Typhimurium* infection can be exploited by this pathogen to suppress the commensal bacterial flora by induction of antimicrobial peptides responsible for metal ion starvation. A strong impact of antimicrobials induced by Stat3 activation on the gut flora is also supported by our study as unlike control mice, *C*. *rodentium* sensitive Stat3^ΔIEC^ mice showed no induction of RegIIIγ, RegIIIβ and Pla2g2a after infection. Remarkably, expression of RegIIIγ and RegIIIβ was completely diminished in Stat3^ΔIEC^ mice, indicating that Stat3 is a key regulator of these AMPs. Although these data might indicate impaired killing of *C*. *rodentium* in Stat3^ΔIEC^ mice, an alternative hypothesis is that *C*. *rodentium* in Stat3^ΔIEC^ mice can capture ecological niches that might have developed from altered AMP expression in Stat3 deficient mice, similarly to the study by Behnsen and colleagues [25]. In support of the hypothesis, Zheng and coworkers demonstrated that administration of exogenous mouse or human RegIIIγ, which acts on gram-positive bacteria [26], to IL-22 deficient mice infected with gram-negative *C*. *rodentium*, partly protected these mice from gastroenteritis [14]. Interestingly, some patients with inflammatory bowel disease also show decreased expression of some AMPs [27]. However, it is still not clear, whether impaired AMP expression is cause or consequence of the disease. Thus, the present study suggests that at least for infections with gastrointestinal pathogens in mice, deficiency in the production of AMPs predispose to a severe course of the disease.

We hypothesize that the altered expression of antimicrobial peptides in the gut of Stat3^ΔIEC^ mice favored *C*. *rodentium* overgrowth and further might facilitate deep penetration of bacteria into the crypt lumen. Indeed, Stat3^ΔIEC^ mice showed abundant bacterial invasion into the lower crypt compartment. Usually, the crypt lumen constitutes a rather bacterial free environment [28] whose colonization would cause significant dangers. Our data support this conclusion since spread of *C*. *rodentium* to distant organs followed by a septic phenotype was observed in Stat3^ΔIEC^ mice after infection. Since *C*. *rodentium* is described as a non-invasive pathogen [23], we suggested death of Stat3 deficient IECs to cause breakdown of the intestinal epithelial barrier integrity allowing invasion of *C*. *rodentium*. However, since increased apoptosis was only detected in infected but not unchallenged Stat3^ΔIEC^ mice, increased death of Stat3 deficient intestinal epithelial cells might only occur under challenged conditions such as *C*. *rodentium* infection. In fact, *C*. *rodentium* has been described to induce cell death in intestinal epithelial cell lines [22].

**Fig 5:**
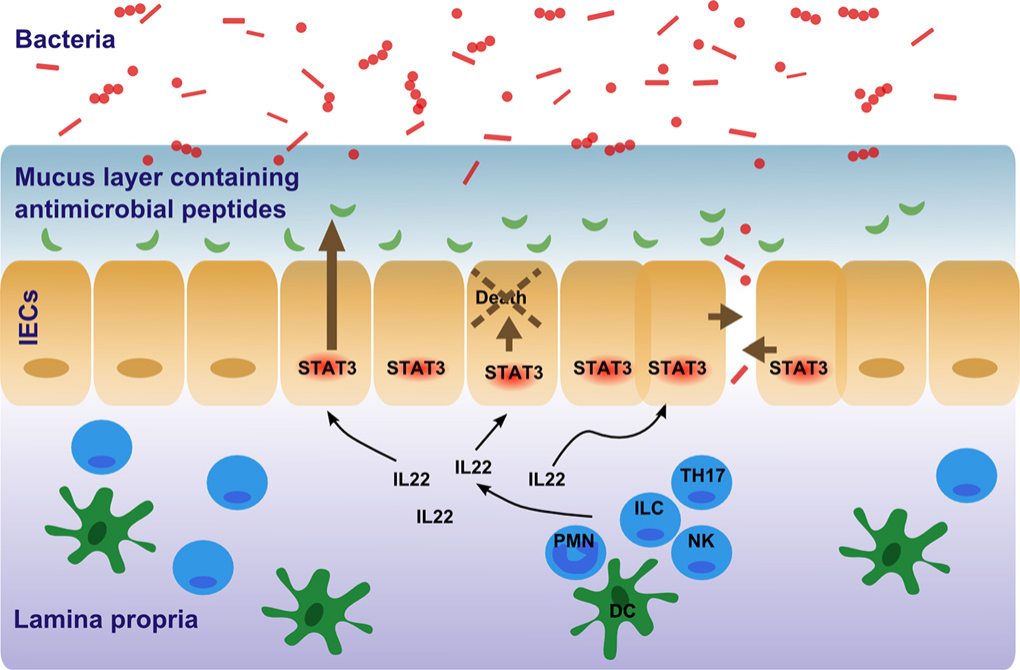
Model of Stat3 activation in intestinal epithelial cells controlling intestinal infections. Activation of Stat3 in intestinal epithelial cells controls bacterial growth and prevents systemic spread of bacteria by regulating the transcription of AMPs and maintaining intestinal epithelial barrier function. Black arrows indicate secretion by and effect of IL-22 on cells. Orange arrows indicate effects of Stat3 activation. DC = dendritic cell, IECs = intestinal epithelial cells, IL-22 = Interleukin-22, ILC = innate lymphoid cells, NK = natural killer cell, TH17 = T helper cell 17, DC = dendritic cell, PMN = polymorphonuclear neutrophil.

In conclusion, we showed that activation of Stat3 in the intestinal epithelium is a key regulator during gastrointestinal infection. Stat3 activation ameliorates intestinal inflammation and prevents systemic spreading of bacteria by controlling growth of bacteria via regulation of AMP expression and by maintaining intestinal epithelial barrier function (Fig. 5). Thus, Stat3 orchestrates the epithelial response to gastrointestinal infections on multiple levels.
